# 超高效液相色谱-三重四极杆质谱法测定水中11大类145种药品和个人护理品

**DOI:** 10.3724/SP.J.1123.2023.04004

**Published:** 2024-01-08

**Authors:** Huijing SUN, Beibei ZHANG, Dongni CUI, Bingjie DONG, Hui WANG, Guanjiu HU

**Affiliations:** 1.江苏省环境监测中心, 国家环境保护地表水环境有机污染物监测分析重点实验室, 江苏 南京 210019; 1. State Environmental Protection Key Laboratory of Monitoring and Analysis for Organic Pollutants in Surface Water, Jiangsu Provincial Environmental Monitoring Center, Nanjing 210019, China; 2.江苏省南京环境监测中心, 江苏 南京 210019; 2. Nanjing Environmental Monitoring Center of Jiangsu Provincial, Nanjing 210019, China

**Keywords:** 超高效液相色谱-三重四极杆质谱, 大体积直接进样, 药品和个人护理品, 环境水体, ultra-high performance liquid chromatography-triple quadrupole mass spectrometry (UHPLC-MS/MS), large volume direct injection, pharmaceuticals and personal care products (PPCPs), environmental water

## Abstract

药品和个人护理品(PPCPs)在水环境中频繁检出,是一类受到国内外环保部门普遍关注的新污染物。准确掌握水环境中PPCPs存在的种类及浓度水平,获取水环境中各类PPCPs的基础污染数据,是环境管理部门进行新污染物管控的重要基础。为此,必须开发准确灵敏、方便快捷且能实现高通量筛查及定量分析的检测方法。本研究采用大体积直接进样-超高效液相色谱-三重四极杆质谱法测定水环境中11大类(抗生素、降压药、降糖药、抗病毒药、*β*-受体激动剂、硝基咪唑类药物、H_2_受体拮抗剂、精神麻醉类药物、降血脂药、非甾体抗炎药及其他类药物和杀菌剂类个人护理品)145种PPCPs。水样经0.22 μm的再生纤维素滤膜过滤,加入乙二胺四乙酸二钠(Na_2_EDTA)并调节pH值至6.0~8.0,加入内标混匀后,采用Phenomenex Kinetex C18柱(50 mm×3 mm, 2.6 μm)进行色谱分离,以含5 mmol/L甲酸铵的0.1%甲酸水溶液-乙腈为正离子扫描模式下的流动相、5 mmol/L甲酸铵水溶液-乙腈为负离子扫描模式下的流动相,分别进行梯度洗脱,质谱智能化分时间段-多反应选择离子监测(Schedule-MRM)模式检测,内标法定量。145种PPCPs在一定的范围内线性良好,方法检出限为0.015~5.515 ng/L,在低、中、高3个加标水平下的回收率为80.4%~128%,相对标准偏差为0.6%~15.6%。将该方法应用于11份地表水样品和6份饮用水样品的检测,结果显示,145种PPCPs中共检出93种化合物,地表水中PPCPs总含量为276.9~2705.7 ng/L,其中抗病毒药、降糖药和精神麻醉类药品的检出率为100%且含量占比最大;饮用水中检出的PPCPs总含量为140.5~211.5 ng/L,主要检出抗生素、抗病毒药和降糖药。该方法简单快捷,可实现上百种PPCPs的同时测定,适用于环境水体中多种PPCPs残留的测定。

## Abstract (en)

Pharmaceuticals and personal care products (PPCPs) are emerging contaminants frequently detected in aquatic environments at trace levels. These chemicals have diverse structures and physicochemical properties and includes pharmaceuticals like antibiotics, antihypertensive drugs, antiviral drugs, and psychotropic drugs that are widely used in large quantities worldwide. Considering the large number of pharmaceuticals currently in usage, it is crucial to establish a priority list of PPCPs that should be monitored and/or treated first. An accurate understanding of the occurrence and levels of PPCPs in aquatic environments is essential for providing objective materials for monitoring these emerging contaminants. Therefore, accurate, efficient, sensitive, and high-throughput screening techniques need to be established for determining and quantifying PPCPs.

This study developed a method for the determination of 145 PPCPs (grouped into eleven categories: antibiotics, antihypertensive drugs, antidiabetic drugs, antiviral drugs, *β*-receptor agonists, nitroimidazoles, H_2_ receptor antagonists, psychotropic drugs, hypolipidemic drugs, non-steroidal anti-inflammatory drugs, and others) in water. The method was based on large volume direct injection without sample enrichment and cleanup and used ultra-high performance liquid chromatography-triple quadrupole mass spectrometry (UHPLC-MS/MS). Water samples were collected and filtered through a 0.22-μm regenerated cellulose (RC) filter membrane. Subsequently, Na_2_EDTA was added to the samples to adjust their pH to 6.0-8.0. Internal standards were mixed with the solutions, and because of the addition of Na_2_EDTA, the interference of metal ions could be eliminated in the determination of compounds, especially for tetracycline and quinolone antibiotics. Among the six filter membranes tested in this study (PES, PFTE-Q, PFTE, MCE, GHP, and RC), RC filter membranes were screened for water sample filtration. The UHPLC-MS/MS parameters were optimized by comparing the results of various mobile phases, as well as by establishing the best instrumental conditions. The 145 PPCPs were separated using an Phenomenex Kinetex C18 column (50 mm×3 mm, 2.6 μm) via gradient elution. The mobile phases were 0.1% (v/v) formic acid aqueous solution containing 5 mmol/L ammonium formate and acetonitrile for positive ion modes, 5 mmol/L aqueous solutions of ammonium formate and acetonitrile for negative ion modes. The samples were quantified using the scheduled multiple reaction monitoring (scheduled-MRM) mode with electrospray ionization in positive and negative ion modes. A standard internal calibration procedure was used to calculate contents of sample. The established method was systematically verified, and it demonstrated a good linear relationship. The average recoveries of the 145 PPCPs at the three spiked levels were in the range of 80.4%-128% with relative standard deviations (RSDs, *n*=6) of 0.6%-15.6%. The method detection limits (MDLs) ranged from 0.015 to 5.515 ng/L. Finally, the optimization method was applied to analyze the 145 PPCPs in 11 surface water samples and 6 drinking water samples. Overall, 93 (64%) out of the 145 analytes were detected. The total contents of the PPCPs in surface water samples ranged from 276.9 to 2705.7 ng/L. The detection frequencies of antidiabetic, antiviral, and psychotropic drugs were 100%. The total contents of the PPCPs in drinking water samples ranged from 140.5 to 211.5 ng/L, and antibiotics, antidiabetic drugs, and antiviral drugs comprised the largest proportion of analytes (by mass concentration) in drinking water samples. Our method exhibited high analytical speed and high sensitivity. It is thus suitable for the trace analysis and determination of the 145 PPCPs in environmental water and showed improved detection efficiency for PPCPs in water, indicating that it has a high potential for practical applications. This study can extend technical support for further pollution-level analysis of PPCPs in water and provide an objective basis for environmental management.

药品和个人护理用品(pharmaceuticals and personal care products, PPCPs)属于新污染物中的一类,涵盖了所有人用和兽用的药品,如抗生素、降压药、降糖药、抗病毒药、*β*-受体激动剂等,以及个人护理品包括抗菌剂、驱虫剂和防腐剂等^[1]^。根据文献报道全球多个天然水体和饮用水中检出大量的PPCPs^[2⇓⇓⇓⇓⇓-8]^。天然水体中的PPCPs主要来自于医院、污水厂和养殖场污水的排放^[9,10]^。PPCPs已经被证实对淡水鱼类和无脊椎动物会产生不良影响^[11,12]^,例如咖啡因会影响海马的神经系统,导致海马反应缓慢并逐渐失去行为控制能力;二甲双胍对鱼类有生殖毒性,能够使雄鱼体内卵黄蛋白原基因表达上调;三氯生可以干扰藻类的光合作用和细菌的繁殖等。同时PPCPs在动植物体内产生富集,进而通过食物链进入人体,影响人体健康^[13,14]^。环境中多种PPCPs共存,混合物毒性可能会产生叠加效应^[15]^,导致毒性增强,在低剂量的长期暴露下将会对人类健康和生态环境带来潜在危害。

PPCPs属于新污染物中的一大类,为加强新污染物治理,保障生态环境安全和人体健康,国家出台了《新污染物治理行动方案》,首次将新污染物调查试点监测纳入生态环境监测体系。因此建立覆盖目标物种类较全的水中药品和个人护理品的检测方法,获取水体环境中各类PPCPs的基础污染数据,可为后续生态环境管理部门加强过程控制、减少污染物排放、规范药品使用、构建完善的药品和个人护理品环境管理体系提供技术基础。

PPCPs种类繁多,且环境中水平低,因此建立灵敏度高、覆盖种类全面、多组分同时分析的高通量分析方法是准确掌握水环境中PPCPs污染水平、有效开展防治措施及风险评估的前提和基础。高效液相色谱-串联质谱法(HPLC-MS/MS)可同时分析多种极性或非极性的PPCPs,成为环境中PPCPs测定的主要仪器方法^[16⇓⇓⇓⇓⇓-22]^,如许红睿等^[21]^建立了17种PPCPs的HPLC-MS/MS测定方法,并将其用于苏州市区113份水源水的分析。固相萃取法广泛用于水中有机物的分离富集,是一种常用的PPCPs前处理方法,但该方法采用选择性吸附与选择性洗脱的原理对目标化合物进行富集浓缩,因此测定的化合物种类具有局限性,且前处理过程冗长。本文在前期研究^[23]^基础上,考虑我国PPCPs的生产和使用情况,结合国家和地方标准^[24⇓⇓⇓⇓⇓⇓⇓⇓⇓⇓⇓⇓⇓⇓⇓⇓⇓⇓⇓⇓⇓⇓-47]^以及标准物质的易获得性,拓展了目标物的种类和数量,选取常见的药物及个人护理品,采用大体积直接进样的方式,建立了UHPLC-MS/MS测定地表水中11大类145种PPCPs的方法,该方法避免了复杂的前处理过程,操作简便快速,灵敏度高,同时具备高通量筛查的特点,为水中PPCPs的检测提供了简单快速的解决方法,同时为获取水体环境中各类PPCPs的基础污染数据以及构建完善的药品和个人护理品环境管理体系提供了技术支撑。

## 1 实验部分

### 1.1 仪器和试剂

超高效液相色谱-三重四极杆质谱联用仪(SCIEX Triple Quad 6500,美国Sciex公司); 0.22 μm再生纤维素滤膜(RC,美国Agilent公司);实验用水为Milli-Q超纯水(美国Millipore公司)。

甲醇、乙腈、甲酸、氨水(色谱纯,美国Merck公司);乙二胺四乙酸二钠(Na_2_EDTA,德国CNW公司)。

145种药物及个人护理品(53种抗生素、11种降压药、13种降糖药、6种抗病毒药、9种*β*-受体激动剂、10种硝基咪唑类、2种H_2_受体拮抗剂、10种精神麻醉类药物、1种降血脂药、18种非甾体抗炎药及12种其他类药品和杀菌剂类个人护理品)和45种内标的具体信息见表1和表2。所有标准品均购自上海安谱实验科技有限公司,纯度均大于98.0%。内标均购自德国Dr. Ehrenstorfer公司,纯度均大于98.0%。

**表1:** 145种PPCPs的保留时间和质谱参数

No.	Compound	t_R_/ min	Parent ion (m/z)	Daughter ion (m/z)	Declustering potential/V	Collison energy/eV	IS
	Antibiotics (抗生素)						
1	sulfachloropyridazine (磺胺氯哒嗪)	5.19	285.1	156.1^*^	21	20	sulfamerazine-d_4_
				92.0	21	39	
2	sulfadiazine (磺胺嘧啶)	3.03	251.1	156.0^*^	40	22	sulfamerazine-d_4_
				92.0	40	32	
3	sulfadimethoxine (磺胺间二甲氧嘧啶)	6.28	311.1	108.2^*^	120	47	sulfamethoxazole-d_4_
				156.1	120	28	
4	sulfamerazine (磺胺甲基嘧啶)	4.07	265.2	172.1^*^	70	23	sulfamerazine-d_4_
				156.1	70	23	
5	sulfamethazine (磺胺二甲嘧啶)	4.69	279.2	186.1^*^	45	24	sulfamerazine-d_4_
				124.2	45	33	
6	sulfamethizole (磺胺甲噻二唑)	4.64	271.0	156.2^*^	30	19	sulfamerazine-d_4_
				108.1	30	35	
7	sulfamethoxazole (磺胺甲恶唑)	5.43	254.0	156.1^*^	49	23	sulfamethoxazole-d_4_
				108.1	49	31	
8	sulfathiazole (磺胺噻唑)	3.72	256.1	156.1^*^	38	21	sulfamerazine-d_4_
				108.1	38	31	
9	sulfapyridine (磺胺吡啶)	3.86	250.3	184.3^*^	60	25	sulfamerazine-d_4_
				156.1	60	23	
10	sulfisomidine (磺胺二甲异嘧啶)	3.17	279.2	186.1^*^	70	23	sulfamerazine-d_4_
				124.4	70	30	
11	sulfisoxazole (磺胺异恶唑)	5.7	268.2	156.1^*^	60	20	sulfamethoxazole-d_4_
				113.3	60	19	
12	sulfameter (磺胺对甲氧嘧啶)	4.66	281.2	215.3^*^	60	25	sulfamethoxazole-d_4_
				156.1	60	25	
13	sulfamonomethoxine (磺胺间甲氧嘧啶)	5.1	281.1	156.1^*^	100	25	sulfamethoxazole-d_4_
				215.1	100	25	
14	sulfachlorpyrazin (磺胺氯吡嗪)	6.17	285.2	156.1^*^	120	22	sulfamethoxazole-d_4_
				92.1	120	37	
15	sulfaquinoxaline (磺胺恶喹啉)	6.31	301.1	156.1^*^	120	22	sulfamethoxazole-d_4_
				92.2	120	40	
16	clindamycin (克林霉素)	5.74	425.3	126.2^*^	80	34	clindamycin-d_3_
				377.2	80	27	
17	lincomycin (林可霉素)	3.97	407.2	126.2^*^	120	35	lincomycin-d_3_
				359.2	120	25	
18	oxytetracycline (土霉素)	4.5	461.3	426.2^*^	100	27	tetracycline-d_6_
				443.2	100	19	
19	chlortetracycline (金霉素)	5.36	479.2	444.1^*^	120	31	tetracycline-d_6_
				462.1	120	24	
20	tetracycline (四环素)	4.72	445.2	410.3^*^	120	27	tetracycline-d_6_
				427.3	120	19	
21	doxycycline (强力霉素)	5.67	445.2	410.1^*^	140	34	tetracycline-d_6_
				428.2	140	27	
22	roxithromycin (罗红霉素)	7.61	837.3	679.2^*^	100	30	roxithromycin-d_7_
				158.1	100	38	
23	clarithromycin (克拉霉素)	7.49	748.3	590.4^*^	120	27	roxithromycin-d_7_
				158.2	120	33	
24	erythromycin (红霉素)	6.70	734.3	576.2^*^	80	27	erythromycin-d_3_
				158.3	80	35	
25	anhydro erythromycin A (脱水红霉素)	7.22	716.3	558.3^*^	90	25	erythromycin-d_3_
				158.3	90	40	
26	azithromycin (阿奇霉素)	5.87	749.7	591.5^*^	160	40	azithromycin-d_3_
				573.6	160	47	
27	cefotaxime (头孢噻肟)	4.41	456.0	396.1^*^	60	15	cephalexin-d_5_
				324.0	60	19	
28	cephapirin (头孢匹林)	3.41	424.2	292.2^*^	60	21	cephalexin-d_5_
				152.0	60	29	
29	cefaclor (头孢克洛)	3.58	368.0	174.2^*^	29	20	cephalexin-d_5_
				106.1	29	39	
30	cefazolin (头孢唑啉)	4.57	455.1	323.1^*^	37	15	cephalexin-d_5_
				156.0	37	22	
31	cephalexin (头孢氨苄)	4.05	348.2	174.0^*^	80	23	cephalexin-d_5_
				158.2	80	15	
32	chloramphenicol (氯霉素)	5.87	321.0	152.1^*^	-40	-24	chloramphenicol-d_5_
				257.0	-40	-15	
33	thiamphenicol (甲砜霉素)	4.33	354.0	185.1^*^	-50	-27	thiamphenicol-d_3_
				290.2	-50	-17	
34	florfenicol (氟苯尼考)	5.48	356.0	336.1^*^	-40	-13	chloramphenicol-d_5_
				185.0	-40	-26	
35	oxilinic acid (噁喹酸)	5.95	262.2	244.2^*^	50	25	ofloxacin-d_3_
				216.3	50	41	
36	sparfloxacin (司帕沙星)	5.35	393.2	349.2^*^	50	27	ofloxacin-d_3_
				292.1	50	35	
37	pipemidic acid (吡哌酸)	4.09	304.1	286.1^*^	118	27	ofloxacin-d_3_
				217.2	142	31	
38	cinoxacin (西诺沙星)	5.56	263.3	245.3^*^	60	22	ofloxacin-d_3_
				217.2	60	30	
39	nalidixic acid (萘啶酸)	6.81	233.2	215.3^*^	54	19	ofloxacin-d_3_
				187.0	54	34	
40	ofloxacin (氧氟沙星)	4.59	362.3	318.0^*^	140	26	ofloxacin-d_3_
				261.1	140	38	
41	norfloxacin (诺氟沙星)	4.57	320.1	302.3^*^	140	27	norfloxacin-d_5_
				276.1	140	24	
42	ciprofloxacin (环丙沙星)	4.67	332.1	314.1^*^	90	31	ciprofloxacin-d_8_
				288.3	90	25	
43	lomefloxacin (洛美沙星)	4.81	352.0	265.2^*^	100	32	ofloxacin-d_3_
				308.1	100	23	
44	sarafloxacin (沙拉沙星)	5.26	386.2	342.3^*^	100	25	ofloxacin-d_3_
				299.0	100	38	
45	enrofloxacin (恩诺沙星)	4.93	360.0	316.2^*^	100	27	ofloxacin-d_3_
				245.2	100	36	
46	marbofloxacin (马波沙星)	4.43	363.2	320.1^*^	80	23	ofloxacin-d_3_
				72.2	80	41	
47	fleroxacin (氟罗沙星)	4.55	370.1	326.1^*^	100	27	ofloxacin-d_3_
				352.2	100	30	
48	pefloxacin (培氟沙星)	4.63	334.2	316.1^*^	124	29	ofloxacin-d_3_
				290.1	124	25	
49	enoxacin (依诺沙星)	4.46	321.1	303.4^*^	108	27	ofloxacin-d_3_
				234.3	108	32	
50	orbifloxacin (奥比沙星)	5.02	396.2	352.3^*^	125	26	ofloxacin-d_3_
				378.0	125	30	
51	difloxacin (二氟沙星)	5.31	400.1	382.1^*^	143	31	ofloxacin-d_3_
				356.0	100	29	
52	flumequine (氟甲喹)	7.05	262.2	244.2^*^	100	23	ofloxacin-d_3_
				202.2	100	44	
53	danofloxacin (达氟沙星)	4.81	358.3	340.4^*^	119	32	ofloxacin-d_3_
				314.0	119	26	
	Antihypertensive drugs (降压药)						
54	atenolol (阿替洛尔)	2.44	267.0	190.0^*^	90	30	atenolol-d_7_
				145.1	90	27	
55	clonidine (可乐定)	3.37	230.1	213.0^*^	114	34	clonidine-d_4_
				160.1	114	47	
56	nisoldipine (尼索地平)	9.8	389.1	239.1^*^	40	24	nifedipine-d_6_
				195.3	40	57	
57	prazosin (哌唑嗪)	5.46	384.3	247.2^*^	170	38	rosiglitazone-d_3_
				138.1	170	42	
58	reserpine (利血平)	7.67	609.2	397.2^*^	215	39	reserpine-d_9_
				195.2	215	46	
59	nifedipine (硝苯地平)	8.17	347.2	315.2^*^	41	12	nifedipine-d_6_
				254.2	41	27	
60	amlodipine (氨氯地平)	7.14	409.0	238.1^*^	50	15	nifedipine-d_6_
				294.1	50	14	
61	nitrendipine (尼群地平)	9.17	361.3	315.1^*^	50	25	nifedipine-d_6_
				328.8	50	19	
62	nimodipine (尼莫地平)	9.58	419.2	343.1^*^	40	11	nifedipine-d_6_
				301.3	40	38	
63	felodipine (非洛地平)	5.54	384.3	338.1^*^	38	14	rosiglitazone-d_3_
				352.1	38	14	
64	hydrochlorothiazide (氢氯噻嗪)	3.12	296.0	269.0^*^	-77	-27	hydrochlorothiazide-^13^C-d_2_
				204.9	-77	-32	
	Antidiabetic drugs (降糖药)						
65	metformin (二甲双胍)	0.88	130.0	60.2^*^	30	18	metformin-d_6_
				71.1	30	29	
66	butadiguanidine (丁二胍)	1.51	158.1	60.1^*^	40	19	metformin-d_6_
				116.1	40	34	
67	phenethyldiguanidine (苯乙二双胍)	3.98	206.0	60.2^*^	50	22	rosiglitazone-d_3_
				105.0	50	38	
68	rosiglitazone (罗格列酮)	5.48	358.1	135.3^*^	115	34	rosiglitazone-d_3_
				107.3	115	57	
69	pioglitazone (吡格列酮)	6.35	357.2	134.0^*^	115	36	rosiglitazone-d_3_
				119.3	115	66	
70	repaglinide (瑞格列奈)	9.6	453.1	162.3^*^	80	29	repaglinide-d_5_
				86.3	80	31	
71	glipizide (格列吡嗪)	7.73	446.1	321.3^*^	65	19	glipizide-d_11_
				346.9	65	20	
72	tolbutamide (甲苯磺丁脲)	7.72	271.0	155.0^*^	48	23	glipizide-d_11_
				74.3	48	17	
73	gliclazide (格列齐特)	8.42	324.1	127.3^*^	116	24	gliclazide-d_4_
				110.3	116	27	
74	glibornuride (格列波脲)	8.80	367.2	170.0^*^	93	22	gliclazide-d_4_
				152.3	93	27	
75	glibenclamide (格列本脲)	9.28	495.1	370.2^*^	50	21	gliclazide-d_4_
				169.3	50	46	
76	glimepiride (格列美脲)	9.50	491.1	126.2^*^	161	34	glimepiride-d_5_
				352.9	161	28	
77	gliquidone (格列喹酮)	10.13	528.1	403.1^*^	175	19	glimepiride-d_5_
				386.2	175	30	
	Antiviral drugs (抗病毒药)						
78	amantadine (金刚烷胺)	4.30	152.2	135.2^*^	45	25	amantadine-d_6_
				93.0	45	41	
79	adamantine (金刚乙胺)	5.71	180.4	163.1^*^	50	22	amantadine-d_6_
				121.2	50	34	
80	memantine (美金刚)	5.83	180.3	163.3^*^	50	21	amantadine-d_6_
				107.0	50	34	
81	imiquimod (咪喹莫特)	5.64	240.8	185.3^*^	123	34	amantadine-d_6_
				168.2	123	47	
82	oseltamivir (奥司他韦)	5.95	313.1	166.0^*^	40	27	amantadine-d_6_
				225.3	40	15	
83	morpholin guanidine (吗啉胍)	0.94	172.2	130.2^*^	68	25	amantadine-d_6_
				113.2	68	27	
	β-Receptor agonists (β-受体激动剂)						
84	terbutaline (特布他林)	1.91	226.2	152.2^*^	49	22	clenbutero-d_9_
				125.0	49	17	
85	cimaterol (西马特罗)	2.06	220.3	202.3^*^	23	15	clenbutero-d_9_
				160.3	23	24	
86	salbutamol (沙丁胺醇)	2.02	240.3	222.2^*^	40	15	salbutamol-d_3_
				148.0	40	26	
87	fenoterol (菲诺特罗)	3.77	304.2	135.1^*^	90	24	clenbutero-d_9_
				107.2	90	47	
88	cloprenaline (氯丙那林)	4.62	214.2	196.2^*^	33	18	clenbutero-d_9_
				154.2	33	25	
89	ractopamine (莱克多巴胺)	4.71	302.2	164.1^*^	63	23	clenbutero-d_9_
				107.1	63	51	
90	clenbutero (克伦特罗)	5.12	277.1	203.1^*^	40	23	clenbutero-d_9_
				259.0	40	16	
91	tulobuterol (妥布特罗)	5.08	228.3	154.1^*^	38	24	clenbutero-d_9_
				172.1	38	16	
92	penbutolol (喷布特罗)	7.47	292.3	236.3^*^	65	23	clenbutero-d_9_
				201.1	65	30	
	Nitroimidazoles (硝基咪唑类)						
93	hydroxymethyl nitrazole (羟甲基硝唑)	1.51	188.4	123.2^*^	48	18	metronidazole-d_4_
				126.2	48	24	
94	2-methyl-4-nitroimidazole (2-甲基-4硝基咪唑)	1.48	128.2	82.1^*^	56	24	metronidazole-d_4_
				42.1	56	51	
95	hydroxydimetridazole (羟基二甲硝咪唑)	1.91	158.0	140.0^*^	35	17	metronidazole-d_4_
				55.1	35	24	
96	metronidazole (甲硝唑)	2.21	172.1	128.2^*^	39	20	metronidazole-d_4_
				82.0	39	33	
97	ronidazole (洛硝哒唑)	2.52	201.4	140.2^*^	31	16	metronidazole-d_4_
				55.0	31	24	
98	dimetridazole (二甲硝咪唑)	2.93	142.0	96.0^*^	54	22	clenbutero-d_9_
				81.0	54	36	
99	5-chloro-1-methyl-4-nitroimidazole	3.6	162.1	116.0^*^	44	24	clenbutero-d_9_
	(氯甲硝咪唑)			145.1	44	23	
100	benzimidazole (苯硝咪唑)	4.23	164.0	118.2^*^	46	30	clenbutero-d_9_
				91.2	46	50	
101	hydroxyisopropinidazole (羟基异丙硝唑)	4.54	186.0	168.1^*^	28	19	clenbutero-d_9_
				122.3	28	27	
102	ipronidazole (异丙硝唑)	5.62	170.2	124.2^*^	56	24	clenbutero-d_9_
				109.2	56	34	
	H_2_ receptor antagonist (H_2_受体拮抗剂)						
103	ranitidine (雷尼替丁)	2.74	315.3	176.1^*^	40	24	clenbutero-d_9_
				130.2	40	33	
104	cimetidine (西咪替丁)	2.29	253.3	159.1^*^	48	20	clenbutero-d_9_
				117.2	48	22	
	Psychotropic drugs (精神麻醉类药物)						
105	caffeine (咖啡因)	4.01	195.0	138.0^*^	80	27	caffeine-^13^C_3_
				110.0	80	31	
106	carbamazepine (卡马西平)	6.85	237.3	194.2^*^	135	26	clenbutero-d_9_
				191.9	135	34	
107	diphenhydramine (苯海拉明)	6.50	256.4	167.0^*^	32	20	clenbutero-d_9_
				152.0	32	52	
108	fluoxetine (氟西汀)	7.56	310.0	148.3^*^	50	12	glipizide-d_11_
				44.3	50	56	
109	olanzapine (奥氮平)	4.19	313.4	256.2^*^	100	37	clenbutero-d_9_
				84.2	100	29	
110	papaverine (罂粟碱)	5.80	340.2	202.1^*^	100	35	glimepiride-d_5_
				324.0	100	44	
111	noscapine (那可丁)	5.87	414.1	220.2^*^	80	29	glimepiride-d_5_
				353.2	80	34	
112	thebaine (蒂巴因)	5.26	312.0	58.0^*^	50	38	glimepiride-d_5_
				266.2	50	25	
113	morphine (吗啡)	1.37	286.3	201.1^*^	100	36	glimepiride-d_5_
				185.1	100	42	
114	codeine (可待因)	3.52	300.0	215.3^*^	113	36	glimepiride-d_5_
				199.0	113	41	
	Hypolipidemic drugs (降血脂药)						
115	rosuvastatin (瑞舒伐他汀)	7.83	482.4	258.2^*^	223	46	clenbutero-d_9_
				272.4	223	46	
Non-steriodal antiinflammatory drugs (非甾体抗炎药)							
116	acetaminophen (对乙酰氨基酚)	2.04	152.1	110.0^*^	20	23	acetaminophen-d_3_
				93.0	20	31	
117	sulindac (舒林酸)	7.53	357.1	233.3^*^	120	65	piroxicam-d_3_
				248.1	120	46	
118	loxoprofen (洛索洛芬)	7.46	264.3	117.2^*^	30	45	piroxicam-d_3_
				201.3	30	18	
119	ketoprofen (酮洛芬)	7.94	255.1	209.2^*^	88	21	piroxicam-d_3_
				105.1	88	32	
120	mefenamic acid (甲芬那酸)	9.8	242.2	223.9^*^	35	20	piroxicam-d_3_
				209.2	35	38	
121	nabumetone (萘普酮)	8.97	229.1	171.1^*^	55	26	piroxicam-d_3_
				128.2	55	52	
122	tenoxicam (替诺昔康)	5.75	338.3	121.0^*^	122	27	piroxicam-d_3_
				95.0	122	26	
123	piroxicam (吡罗昔康)	7.12	332.2	95.1^*^	115	23	piroxicam-d_3_
				121.2	115	26	
124	meloxicam (美诺西康)	7.98	352.3	115.1^*^	153	23	piroxicam-d_3_
				141.1	153	27	
125	rofecoxib (罗非昔布)	7.42	315.1	269.0^*^	110	29	piroxicam-d_3_
				192.2	110	33	
126	antipyrine (安替匹林)	4.74	189.1	77.0^*^	95	54	piroxicam-d_3_
				106.1	95	36	
127	phenylbutazone (保泰松)	9.55	309.1	160.3^*^	126	29	phenylbutazone-^13^C
				188.3	126	22	
128	tolmetin (托美汀)	7.66	258.0	119.0^*^	45	50	piroxicam-d_3_
				91.0	45	23	
129	diclofenac (双氯芬酸)	9.14	294.1	250.0^*^	-12	-17	meclofenamic acid-^13^C_6_
				214.0	-12	-27	
130	meclofenamic acid (甲氯芬那酸)	9.88	293.8	258.1^*^	-40	-19	meclofenamic acid-^13^C_6_
				214.1	-40	-27	
131	nimesulide (尼美舒利)	8.55	306.9	229.0^*^	-35	-23	meclofenamic acid-^13^C_6_
				198.0	-35	-38	
132	parecoxib (帕瑞昔布)	7.78	369.1	119.0^*^	-100	-38	meclofenamic acid-^13^C_6_
				234.2	-100	-32	
133	etodolac (依托度酸)	8.07	285.7	212.1^*^	-36	-34	meclofenamic acid-^13^C_6_
				242.1	-36	-26	
Other drugs and personal care product with fungicides (其他类药品及杀菌剂类个人护理品)							
134	cotinine (可替宁)	1.19	177.1	98.1^*^	25	21	cotinine-d_3_
				80.2	133	31	
135	ormetoprim (奥美普林)	4.60	275.2	259.3^*^	145	36	clenbutero-d_9_
				123.2	145	33	
136	diltiazem (地尔硫卓)	6.72	415.2	178.1^*^	80	34	clenbutero-d_9_
				150.2	80	62	
137	fluconazol (氟康唑)	5.09	306.9	238.0^*^	60	20	clenbutero-d_9_
				220.0	60	25	
138	ketoconazole (酮康唑)	7.35	531.0	489.1^*^	50	43	clenbutero-d_9_
			533.0	491.0	50	46	
139	miconazole (咪康唑)	9.06	417.1	159.0^*^	50	36	clenbutero-d_9_
				161.1	50	36	
140	gemfibrozil (吉非罗齐)	10.02	249.3	121.0^*^	-35	-22	gemfibrozil-d_6_
				127.0	-35	-14	
141	warfarin (华法林)	8.52	307.2	161.0^*^	-85	-27	warfarin-d_5_
				250.1	-85	-30	
142	diethyltoluamide (避蚊胺)	7.63	192.1	119.1^*^	80	25	atrazine-d_5_
				91.1	80	40	
143	triclosan (三氯生)	10.54	287.0	35.0^*^	-104	-45	trichlorocarban-^13^C_6_
			289.0	35.0	-104	-45	
144	trichlorocarban (三氯卡班)	10.46	315.0	162.0^*^	-60	-23	trichlorocarban-^13^C_6_
			313.1	160.0	-140	-22	
145	1,7-dimethylxanthine (1,7-二甲基黄嘌呤)	2.78	181.2	124.0^*^	120	28	atrazine-d_5_
				69.2	120	40	

* Quantitative ion.

**表2:** 45种内标的保留时间和质谱参数

Compound	t_R_/min	Parent ion (m/z)	Daughter ion (m/z)	Declustering potential/V	Collison energy/eV
Caffeine-^13^C_3_(咖啡因-^13^C_3_)	4.02	198.0	140.0^*^	80	27
Reserpine-d_9_(利血平-d_9_)	7.65	618.4	397.2	215	38
Gliclazide-d_4_(格列齐特-d_4_)	8.42	328.1	127.3	100	25
Olanzapine-d_30_(奥氮平-d_30_)	4.19	316.0	256.1	100	32
Fluoxetine-d_5_(氟西汀-d_5_)	7.55	315.0	44.0	50	54
Ciprofloxacin-d_8_(环丙沙星-d_8_)	4.70	340.0	322.0	90	31
Glipizide-d_11_(格列吡嗪-d_11_)	7.73	457.2	321.3	44	38
Rosiglitazone-d_3_(罗格列酮-d_3_)	5.50	361.0	138.4	115	36
Enrofloxacin-d_5_(恩诺沙星-d_5_)	4.94	365.0	321.0	100	26
Azithromycin-d_3_(阿奇霉素-d_3_)	5.90	752.7	594.5	160	40
Cotinine-d_3_(可替宁-d_3_)	1.20	180.1	101.0	25	21
Lincomycin-d_3_(林可霉素-d_3_)	4.00	410.2	129.0	120	35
Glibenclamide-d_11_(格列本脲-d_11_)	9.28	505.4	369.3	158	19
Glimepiride-d_5_(格列美脲-d_5_)	9.53	496.2	357.0	158	20
Metformin-d_6_(二甲双胍-d_6_)	0.90	136.3	60.0	36	18
Repaglinide-d_5_(瑞格列奈-d_5_)	9.60	458.1	230.4	84	40
Nifedipine-d_6_(硝苯地平-d_6_)	8.17	353.4	318.1	38	12
Clonidine-d_4_(可乐定-d_4_)	3.35	234.1	217.1	122	36
Salbutamol-d_3_(沙丁胺醇-d_3_)	2.02	243.3	225.1	27	15
Piroxicam-d_3_(吡罗昔康-d_3_)	7.14	335.2	95.0	115	23
Phenylbutazone-^13^C(保泰松-^13^C)	9.56	321.0	166.2	128	30
Acetaminophen-d_3_(对乙酰氨基酚-d_3_)	2.05	155.0	111.3	20	23
Sulfamerazine-d_4_(磺胺甲基嘧啶-d_4_)	4.07	269.2	172.1	70	23
Sulfamethoxazole-d_4_(磺胺甲恶唑-d_4_)	5.43	258.3	160.0	49	23
Ofloxacin-d_3_(氧氟沙星-d_3_)	4.60	365.2	321.0	140	26
Erythromycin-d_3_(红霉素-d_3_)	6.70	738.7	580.3	80	27
Norfloxacin-d_5_(诺氟沙星-d_5_)	4.57	325.3	307.1	85	29
Cephalexin-d_5_(头孢氨苄-d_5_)	4.01	353.0	179.0	80	23
Tetracycline-d_6_(四环素-d_6_)	4.72	451.4	416.2	120	27
Roxithromycin-d_7_(罗红霉素-d_7_)	7.61	844.3	686.2	100	30
Clindamycin-d_3_ (克林霉素-d_3_)	5.74	428.0	128.0	80	34
Clenbutero-d_9_(克伦特罗-d_9_)	5.12	286.2	268.3	40	16
Amantadine-d_6_(金刚烷胺-d_6_)	4.30	158.4	141.3	38	24
Atenolol-d_7_(阿替洛尔-d_7_)	2.44	274.1	190.3	90	30
Metronidazole-d_4_(甲硝唑-d_4_)	2.21	176.1	128.2	39	20
Atrazine-d_5_(阿特拉津-d_5_)	7.44	221.1	101.0	70	26
Tolbutamide-d_9_(甲苯磺丁脲-d_9_)	7.73	280.1	155.0	50	26
Prazosin-d_8_(哌唑嗪-d_8_)	5.47	392.2	251.0	60	42
Meclofenamic acid-^13^C_6_(甲氯芬那酸-^13^C_6_)	9.88	299.8	264.1	-35	-19
Trichlorocarban-^13^C_6_(三氯卡班-^13^C_6_)	10.50	318.9	159.7	-53	-20
Hydrochlorothiazide-^13^C-d_2_(氢氯噻嗪-^13^C-d_2_)	3.12	299.1	269.9	-77	-29
Warfarin-d_5_(华法林-d_5_)	8.52	312.0	161.0	-111	-27
Gemfibrozil-d_6_(吉非罗齐-d_6_)	10.02	255.3	121.0	-34	-33
Chloramphenicol-d_5_(氯霉素-d_5_)	5.87	326.1	157.0	-40	-24
Thiamphenicol-d_3_(甲砜霉素-d_3_)	4.33	359.1	295.0	-50	-18

### 1.2 标准溶液配制

量取100 mL纯水,加入0.025 g Na_2_EDTA,再用甲酸或氨水调节pH值至6.0~8.0,配制成含有Na_2_EDTA的稀释溶液。

标准品用甲醇(头孢噻肟、头孢匹林、头孢克洛、头孢唑啉、头孢氨苄用50%(v/v)乙腈水溶液)配成1000 μg/mL的标准储备液,于-20 ℃冰箱保存。用甲醇配制1 μg/mL的混合标准溶液,使用时用含Na_2_EDTA的稀释溶剂配制成需要的浓度。

45种内标分别用甲醇配制成100 μg/mL的内标储备液;用甲醇进一步稀释配制质量浓度为10.0 ng/mL的内标混合使用液。

### 1.3 样品前处理

根据文献[23]方法进行样品前处理。量取100 mL水样,经0.22 μm的再生纤维素滤膜过滤后,加入0.025 g Na_2_EDTA,再用甲酸或氨水调节pH值至6.0~8.0。取调节后的水样1.0 mL,加入10 μL内标使用液后,混匀,直接进样。

### 1.4 分析条件

#### 1.4.1 色谱条件

色谱柱:Phenomenex Kinetex C18柱(50 mm×3 mm, 2.6 μm,美国菲罗门公司);正离子模式:流动相A为含5 mmol/L甲酸铵的0.1%甲酸水溶液,流动相B为乙腈;负离子模式:流动相A为5 mmol/L甲酸铵水溶液,流动相B为乙腈;流速:0.4 mL/min;梯度洗脱程序:0~1.5 min, 5%B; 1.5~10.0 min, 5%B~70%B; 10.0~13.0 min, 70%B~90%B; 13.0~14.0 min, 90%B; 14.0~14.2 min, 90%B~5%B; 14.2~15.0 min, 5%B。进样量:100 μL。

#### 1.4.2 质谱条件

采用ESI源,离子源加热温度为500 ℃,检测方式为智能化分时间段-多反应选择离子监测(Schedule-MRM)模式。正离子/负离子检测;喷雾电压为5500 V/-4500 V;雾化气压力为345 kPa (50 psi);辅助气压力为345 kPa (50 psi);气帘气压力为207 kPa (30 psi)。各化合物的质谱参数见表1。

## 2 结果与讨论

### 2.1 质谱条件的优化

配制145种药品和个人护理品及其内标混合溶液(100 μg/L),根据化合物的化学性质及结构,分别在ESI源正、负离子扫描模式下采用流动注射进入质谱进行扫描,确定最佳去簇电压、碰撞能量及各化合物的母离子和子离子等质谱参数,优化后的质谱条件见表1和表2。

### 2.2 色谱条件的优化

在ESI^+^模式下为了保证化合物更好的质子化,实验比对了0.1%甲酸水溶液-乙腈和含5 mmol/L甲酸铵的0.1%甲酸水溶液-乙腈作为流动相对目标物灵敏度和分离度的影响。结果表明,用含5 mmol/L甲酸铵的0.1%甲酸水溶液作为水相时,化合物的响应更高,分离更好,因此选用含5 mmol/L甲酸铵的0.1%甲酸水溶液作为正离子扫描模式下的水相。在ESI^-^模式下,实验对比了水-乙腈和5 mmol/L甲酸铵水溶液-乙腈作为流动相的效果,结果表明以水-乙腈为流动相时,部分化合物的色谱峰形差且响应低,以双氯芬酸、尼美舒利、帕瑞昔布、依托度酸为例,它们在该流动相下出现色谱峰前延、分叉、峰展宽等现象,加入5 mmol/L甲酸铵以后,峰形得到明显的改善,色谱峰尖锐对称(见图1),故选择5 mmol/L甲酸铵水溶液-乙腈作为ESI^-^模式下的流动相。最终确定ESI^+^模式下以含5 mmol/L甲酸铵的0.1%甲酸水溶液-乙腈为流动相,ESI^-^模式下以5 mmol/L甲酸铵水溶液-乙腈为流动相,145种化合物能实现较好的分离(见图2)。

**图1:**
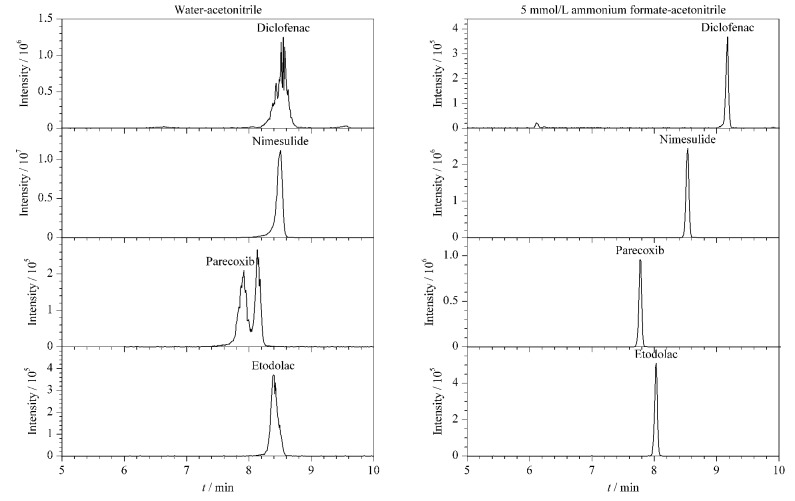
不同流动相条件下4种PPCPs的色谱图

**图2:**
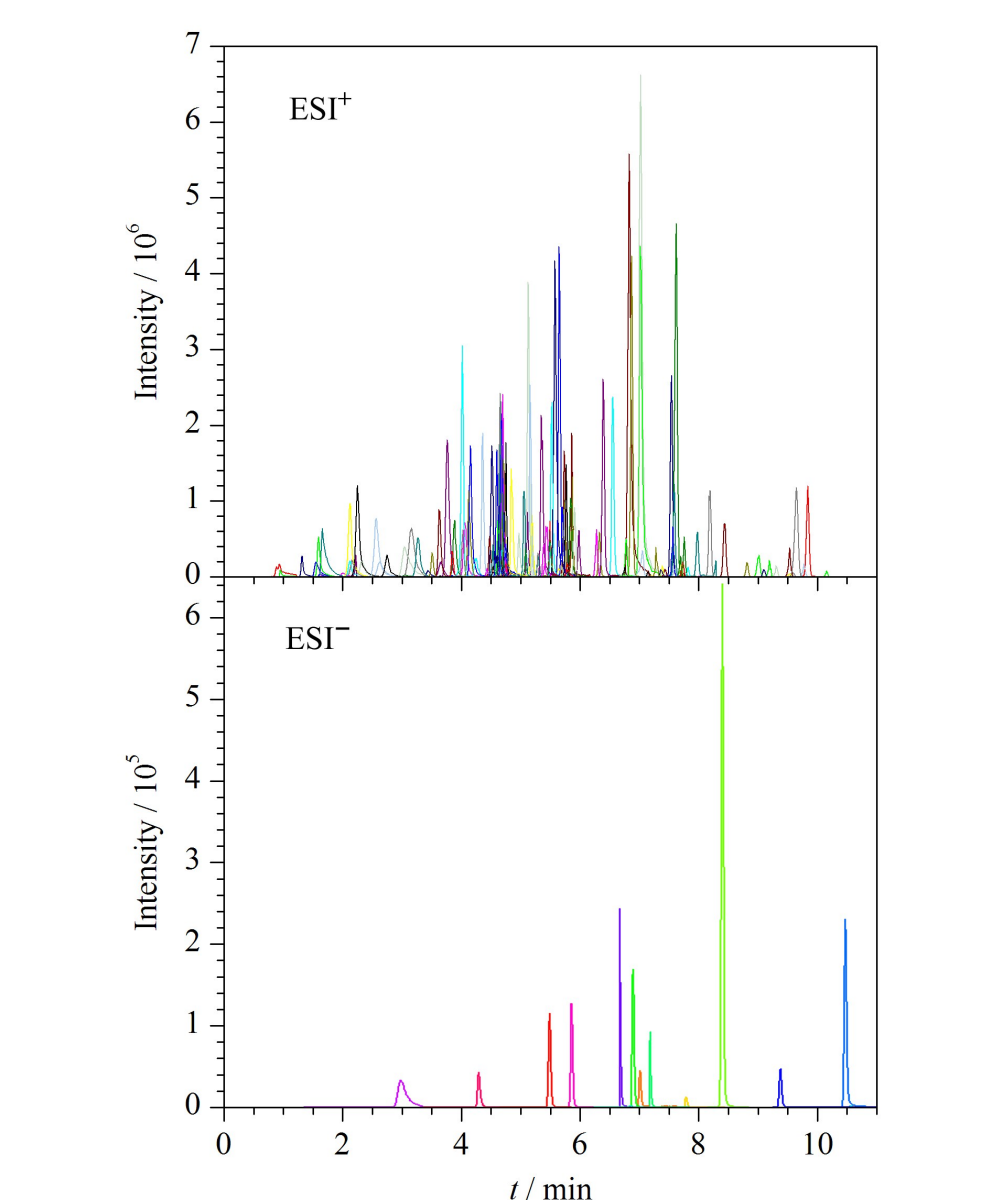
145种PPCPs在正、负离子模式下的总离子流色谱图

### 2.3 Na_2_EDTA的影响

PPCPs中含F、N、O、S等富电子的基团种类较多,在水中与金属共存时易发生络合反应形成化合物-金属离子络合物。例如许多抗生素都含有羧基、羰基或哌嗪基等官能团,它们能充当潜在的电子供体与金属配位^[48,49]^。另有研究^[50]^表明,金属离子所带电荷数越高,与抗生素的络合倾向越大。Na^+^和K^+^等一价金属离子基本无络合能力,Ca^2+^和Mg^2+^具有较强的络合能力,Fe^3+^和Cu^2+^的络合能力最强。所以本研究选用含有Ca^2+^(30.2 mg/L)、Mg^2+^(8.9 mg/L)、Cu^2+^(0.01 mg/L)、Fe^3+^(0.01 mg/L)的地表水为研究对象,选择Na_2_EDTA作为金属离子抑制剂,考察Na_2_EDTA的加入对各类PPCPs回收率的影响(见图3),具体回收率数据见附表1(详见 https://www.chrom-China.com)。

**图3:**
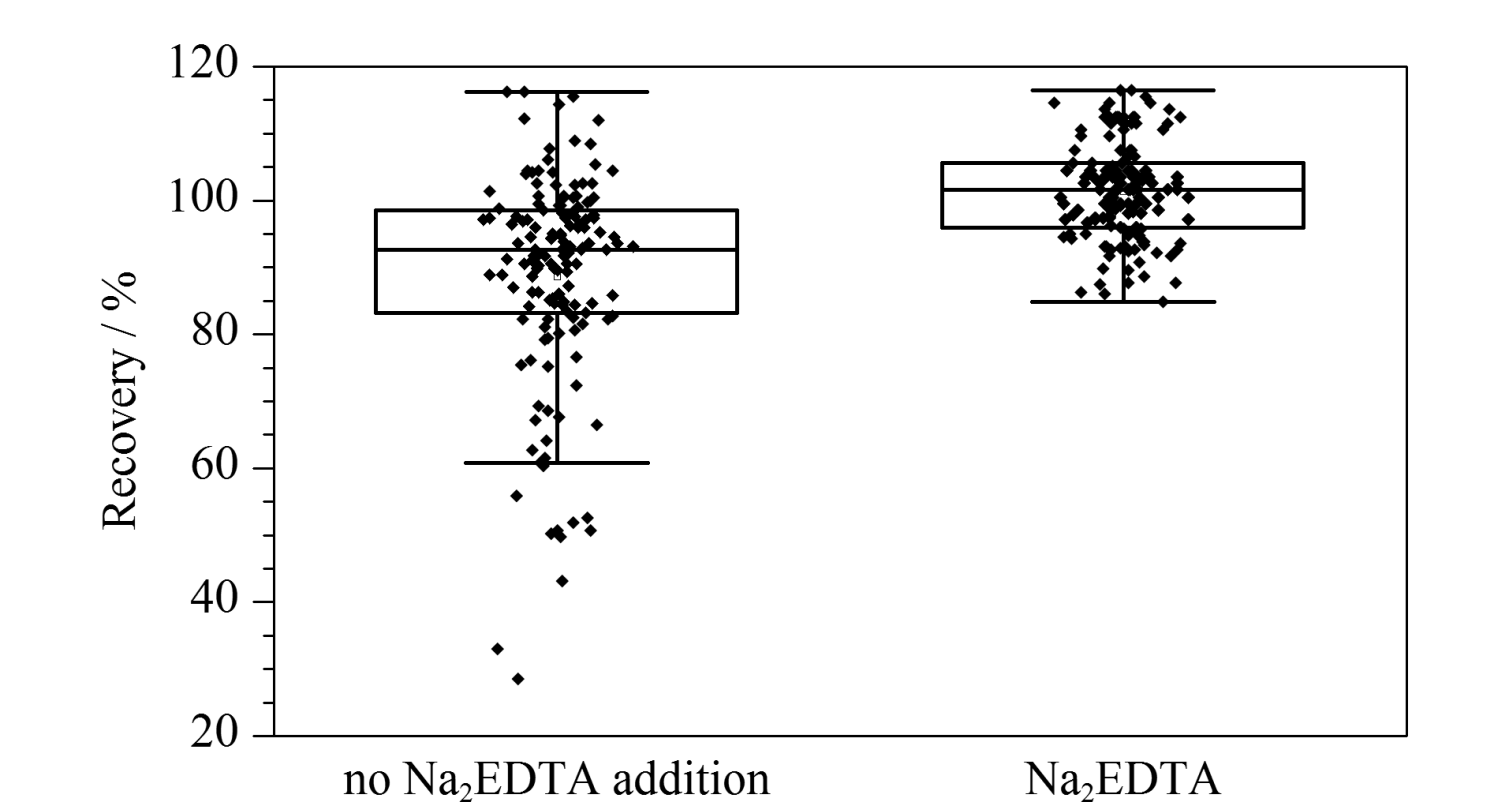
Na_2_EDTA添加对地表水样品中145种PPCPs回收率的影响

结果显示,未添加和添加Na_2_EDTA对大部分化合物回收率的影响不大,但对一部分化合物而言,添加Na_2_EDTA后回收率明显升高,例如降压药尼索地平的回收率从61.2%提高至98.1%;罗红霉素、克拉霉素等大环内酯类抗生素的回收率从43.6%~66.6%提升至95.3%~113%;司帕沙星、吡哌酸等喹诺酮类抗生素的回收率从50.3%~66.9%提升至93.2%~96.3%;金霉素、四环素等四环素类抗生素的回收率从52.2%~69.7%提升至90.1%~112%。虽然受金属干扰测定的化合物数量不多,但它们都是在环境中检出率较高的几类抗生素,因此选择在水样中加入Na_2_EDTA,以保证各类化合物能够被准确测定。

### 2.4 滤膜的选择

选择了6种孔径为0.22 μm,材质分别为聚醚砜(PES)、亲水性聚四氟乙烯(PTFE-Q)、聚四氟乙烯滤膜(PTFE)、混合纤维素滤膜(MCE)、聚丙烯滤膜(GHP)和再生纤维素的一次性针式过滤器,对100 ng/L的空白加标样品进行分析,所有组分的加标回收率如图4所示,结果显示,材质为RC时目标化合物的平均回收为84.4%~116%,均能满足检测要求,故选择RC滤膜过滤水样。

**图4:**
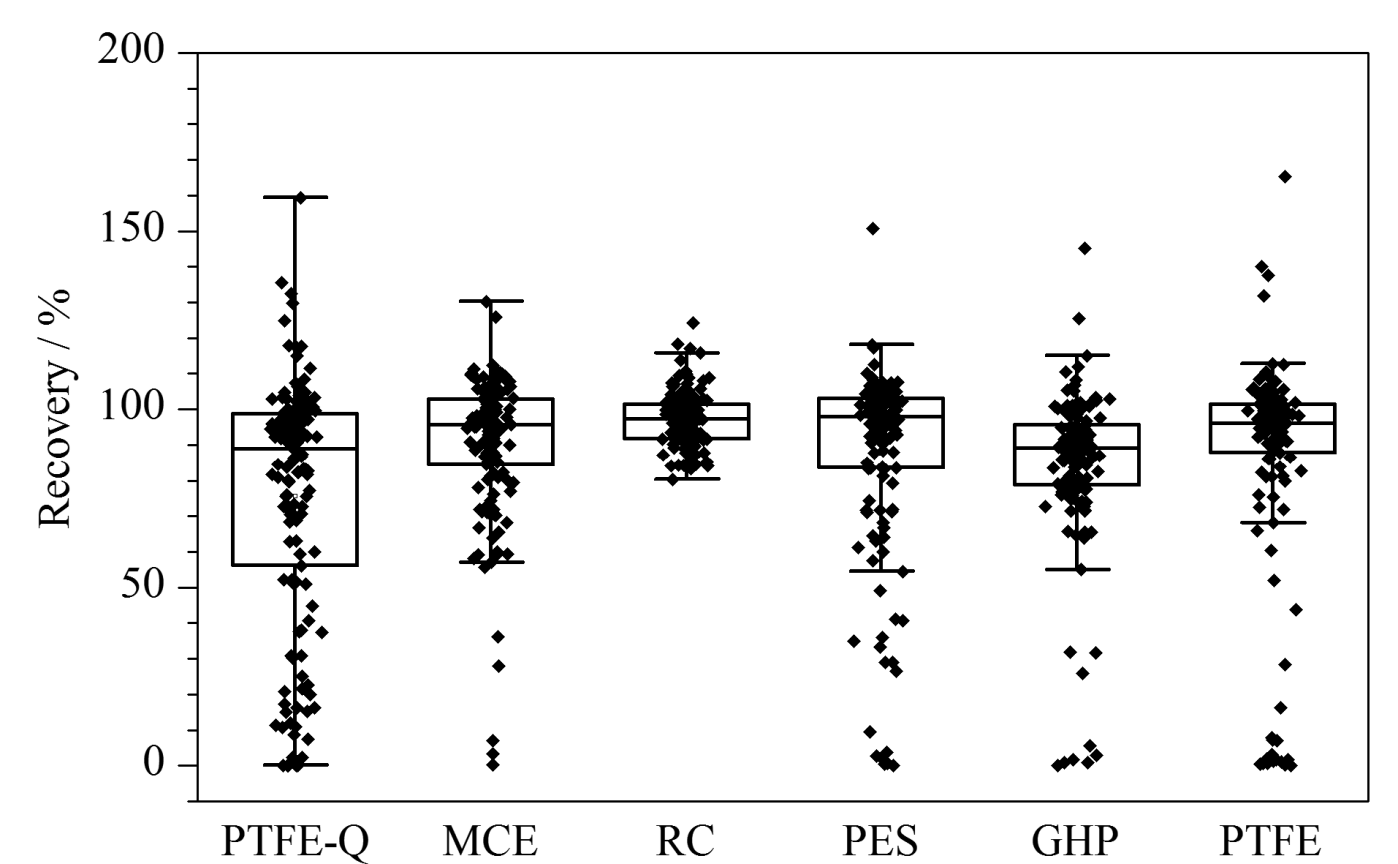
不同滤膜材质对145种PPCPs回收率的影响

### 2.5 方法学验证

#### 2.5.1 线性范围与方法检出限

配制每种化合物的系列混合标准溶液,按确定的分析条件进行测定,以各物质的质量浓度(ng/L)为横坐标*X*,以其对应的峰面积与内标峰面积的比值为纵坐标*Y*,绘制标准曲线。结果显示,145种PPCPs在各自的线性范围内线性良好(见附表2)。

根据HJ 168-2020方法检出限的测定要求,将145种低浓度PPCPs标准溶液添加至超纯水中,按照样品分析过程平行测定7份。结果表明,145种PPCPs的方法检出限为0.015~5.515 ng/L(见附表2)。方法灵敏度高,完全能够满足环境水体中PPCPs检测的要求。

#### 2.5.2 准确度与精密度

分别于实验用水和空白地表水中加入低、中、高3种水平的混合标准工作液,每个水平各平行测定6次,考察其回收率及相对标准偏差。结果表明,方法的回收率为80.4%~128%,相对标准偏差为0.6%~15.6%(见附表3)。

### 2.6 与其他方法的比较

现有的分析方法能测定水中十几至上百种PPCPs,对于种类繁多的PPCPs而言,很难找到适合所有分析物的最佳pH值,因此前处理大多在不同pH值条件下进行固相萃取。目前EPA 1694^[51]^是PPCPs类物质的标准分析方法,该方法使用固相萃取对水中74种PPCPs进行前处理,范围涵盖了抗生素、降压药、降血脂药等多种药物类别,是非常全面的分析方法,但该方法需要经过2种不同的前处理过程和4种不同的仪器分析方法,整个分析方法耗时长,步骤繁琐,方法不容易普及。因此,亟须建立操作简单、易普及、测定结果可靠并且可以同时测定多种PPCPs的分析方法。本文方法与文献报道和标准方法相比(见表3),灵敏度可以满足痕量测定的要求,回收率良好,大大节约了前处理时间,能够达到快速分析及高通量筛查的目的,为环境水体中PPCPs的监测提供快速、高效、灵敏的分析方法。

**表3:** 与其他方法的比较

Compounds	Procedure	Recoveries/%	LODs/(μg/L)	MDLs/(ng/L)	Ref.
145 PPCPs	large volume direct injection	80.4-128	/	0.015-5.515	this method
23 PPCPs	SPE at pH=3 and pH=4	22.7-127.6	/	0.227-33.3	[52]
70 PPCPs	SPE at pH=2.5 and pH=6.5	45-168	/	1-206	[53]
42 PPCPs	SPE at pH=2.5	9.1-1946.4	0.01-0.1	/	[54]
168 PPCPs and metabolites	SPE at pH=3 and pH=7	77-117	/	0.01-2.61	[55]
18 PPCPs	SPE at pH=3 and no pH adjustment	49-120	/	/	[56]
12 PPCPs	SPE with no pH adjustment	61-110	/	0.02-1.5	[20]

MDL: method detection limit.

### 2.7 实际样品的分析

采用本文建立的方法对20个地表水样品和6个饮用水样品进行测定,共有93种(64%)化合物被检出,结果见附表4和附表5。

20个地表水样品中PPCPs总含量为276.9~2705.7 ng/L,平均含量为989.2 ng/L。其中抗病毒药、降糖药和精神麻醉类药品检出的含量所占比例最大(见图5),检出率为100%,含量最高的化合物分别为金刚烷胺(771.4 ng/L)、二甲双胍(632.8 ng/L)和咖啡因(452.6 ng/L)。6个饮用水样品中检出的PPCPs总含量为140.5~211.5 ng/L,平均含量为177.8 ng/L,主要检出抗生素、抗病毒药和降糖药(见图5)。

**图5:**
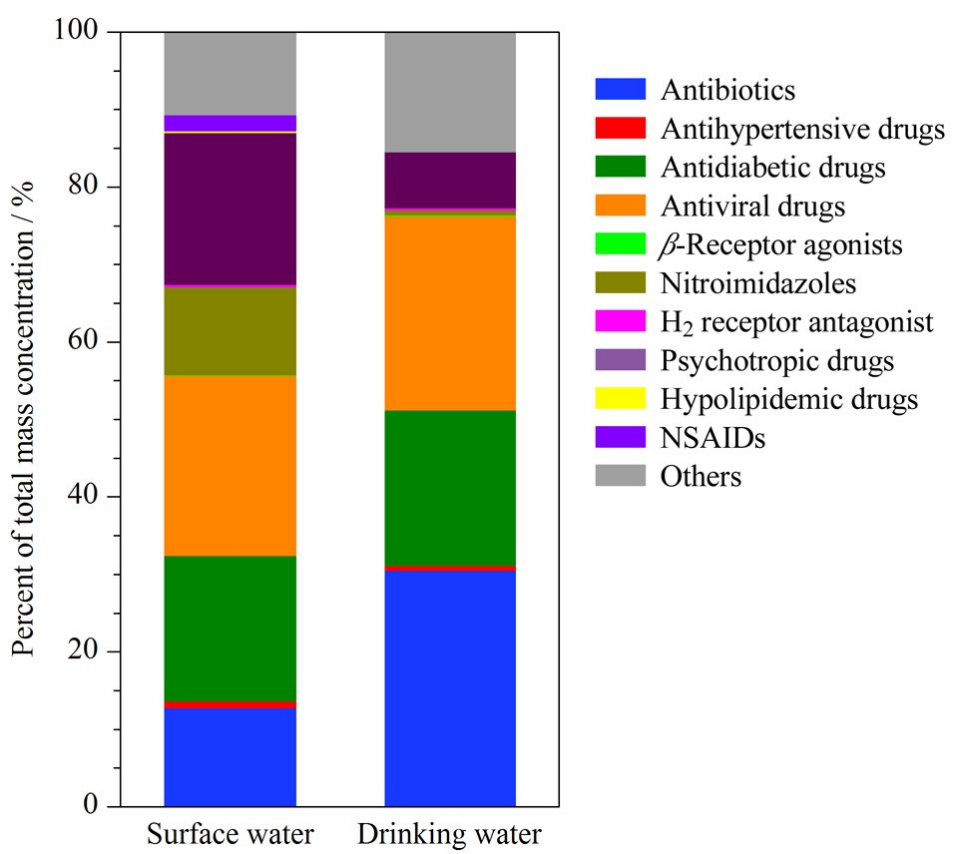
地表水和饮用水中11大类PPCPs的总含量占比

## 3 结论

本文建立了大体积直接进样测定水环境中145种PPCPs的超高效液相色谱-三重四极杆质谱法。方法无需对水样富集,精密度好,灵敏度高,操作方便,实用性强,可为大批量同时快速检测地表水和生活饮用水中145种PPCPs提供技术参考,为准确掌握水体中PPCPs的污染水平及环境管理提供技术支撑。
